# Localization and site-specific cell–cell interactions of group 2 innate lymphoid cells

**DOI:** 10.1093/intimm/dxab001

**Published:** 2021-01-06

**Authors:** Tsuyoshi Kiniwa, Kazuyo Moro

**Affiliations:** 1 Laboratory for Innate Immune Systems, RIKEN Center for Integrative Medical Sciences (IMS), 1-7-22 Suehiro-cho, Tsurumi-ku, Yokohama, Kanagawa, Japan; 2 Laboratory for Innate Immune Systems, Department of Microbiology and Immunology, Graduate School of Medicine, Osaka University, 2-2 Yamadaoka Suita-shi, Osaka, Japan; 3 Laboratory for Innate Immune Systems, IFReC, Osaka University, 3-1 Yamadaoka Suita-shi, Osaka, Japan

**Keywords:** ASCs, ILC2s, microenvironment, neurons, WAT-MSCs

## Abstract

Group 2 innate lymphoid cells (ILC2s) are novel lymphocytes discovered in 2010. Unlike T or B cells, ILC2s are activated non-specifically by environmental factors and produce various cytokines, thus playing a role in tissue homeostasis, diseases including allergic diseases, and parasite elimination. ILC2s were first reported as cells abundantly present in fat-associated lymphoid clusters in adipose tissue. However, subsequent studies revealed their presence in various tissues throughout the body, acting as key players in tissue-specific diseases. Recent histologic analyses revealed that ILC2s are concentrated in specific regions in tissues, such as the lamina propria and perivascular regions, with their function being controlled by the surrounding cells, such as epithelial cells and other immune cells, via cytokine and lipid production or by cell–cell interactions through surface molecules. Especially, some stromal cells have been identified as the niche cells for ILC2s, both in the steady state and under inflammatory conditions, through the production of IL-33 or extracellular matrix factors. Additionally, peripheral neurons reportedly co-localize with ILC2s and alter their function directly through neurotransmitters. These findings suggest that the different localizations or different cell–cell interactions might affect the function of ILC2s. Furthermore, generally, ILC2s are thought to be tissue-resident cells; however, they occasionally migrate to other tissues and perform a new role; this supports the importance of the microenvironment for their function. We summarize here the current understanding of how the microenvironment controls ILC2 localization and function with the aim of promoting the development of novel diagnostic and therapeutic methods.

## Teaser

Microenvironmental regulation of ILC2 functions

## Introduction

Group 2 innate lymphoid cells (ILC2s) are defined as lymphoid cells lacking specific lineage markers and expressing GATA-binding protein 3 (GATA3) (1). Unlike adaptive lymphocytes, ILC2s lack rearranged antigen receptors, such as the T-cell antigen receptor and B-cell antigen receptor; they cannot directly respond to exogenous antigens. Instead, they strongly and rapidly react to environmental factors, including cytokines, lipid mediators, neurotransmitters, hormones and nutrients (Table 1). In response to these factors, ILC2s reportedly produce various cytokines, including IL-4, IL-5, IL-6, IL-9, IL-13, GM-CSF and amphiregulin, and are associated with the pathogenesis of or protection against various diseases, such as parasite infections, allergies, fibrosis, autoimmune diseases, obesity, sepsis and cancer (2–10).

**Table 1.** Summary of the regulatory factors from tissue-constituting cells

	Molecule	Style	Effect
Epithelial cell (EC)			
Common EC	IL-33	Humoral	Activation
	TSLP	Humoral	Activation
	TNF-like cytokine 1A	Cell–cell contact	Activation
	E-cadherin	Cell–cell contact	Inhibition
Tuft cell	IL-25	Humoral	Activation
	LTC4	Humoral	Activation
PNEC	CGRP	Humoral	Activation/inhibition
Neurons			
Sensory neuron	VIP	Humoral	Activation
Sympathetic neuron	NE	Humoral	Inhibition
Parasympathetic neuron	NMU	Humoral	Activation
	CGRP	Humoral	Activation/inhibition
Immune cells			
T_h_2 cell	IL-4	Humoral	Activation
	IL-9	Humoral	Activation
T_reg_	IL-10	Humoral	Inhibition
	TGF-13	Humoral	Inhibition
DCs	IL-27	Humoral	Inhibition
cDC2	IL-4	Humoral	Activation
	IL-33	Humoral	Activation
	GITR ligand	Cell–cell contact	Activation
pDC	Type 1 interferon	Humoral	Inhibition
M1 macrophage	PD-L1	Cell–cell contact	Inhibition
M2 macrophage	PGD2	Humoral	Activation
	LTs	Humoral	Activation
Basophil, mast cell	IL-2	Humoral	Activation/inhibition
	IL-4	Humoral	Activation
	IL-33	Humoral	Activation
	Prostaglandins	Humoral	Activation
	Leukotrienes	Humoral	Activation
ILC2s	IL-2	Humoral	Activation
	IL-9	Humoral	Activation
	ICOS ligand	Cell–cell contact	Activation
	PD-L1	Cell–cell contact	Inhibition
Other cells			
ASCs	IL-33	Humoral	Activation
	TSLP	Humoral	Activation
WAT-MSCs	IL-33	Humoral	Activation
	ICAM-1	Cell–cell contact	Activation
Tumor cells	Lactic acid	Humoral	Inhibition
	PD-L1	Cell–cell contact	Inhibition

**Fig. 1.**
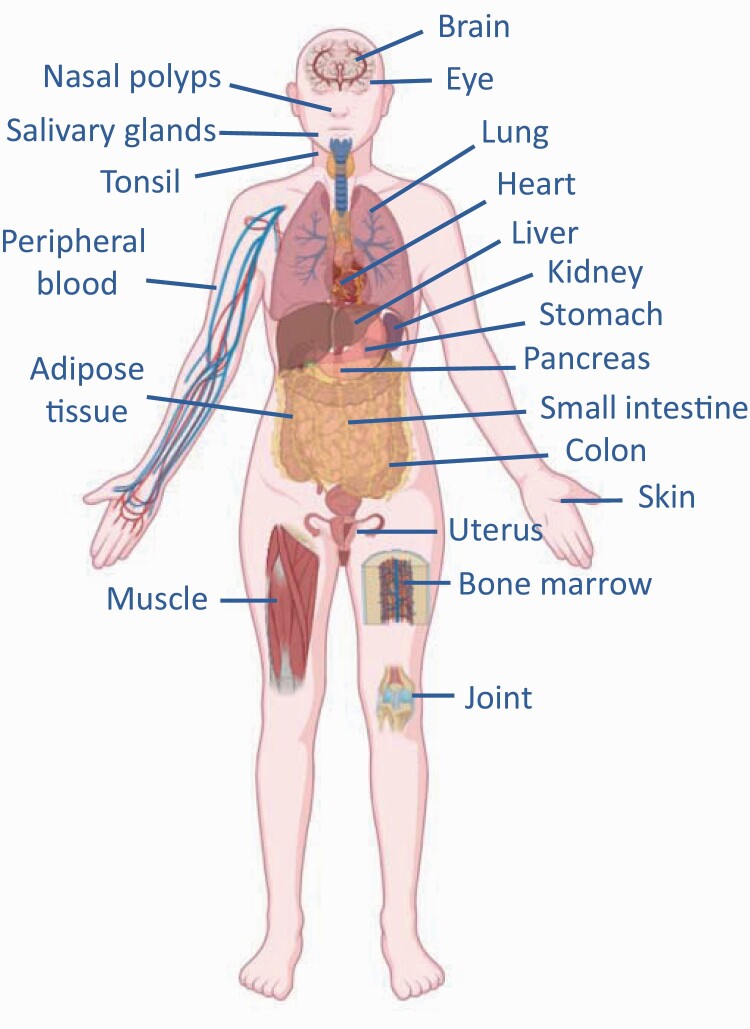
ILC2s reside in many tissues. Human and mouse studies clarified that ILC2s exist in various tissues in the physiological steady state. ILC2s possess tissue-specific functions and play a pivotal role in maintaining the homeostasis of tissues wherein they reside.

ILC2s are tissue-resident cells that exist in peripheral tissues during the steady state and produce type 2 cytokines constitutively to maintain tissue homeostasis by regulating the expansion and survival of B1 cells or eosinophils by producing small amounts of IL-5 (2, 11). Moreover, in response to tissue injury (with or without inflammation), ILC2s contribute to tissue regeneration by enhancing the differentiation and expansion of epithelial cells by the production of IL-13 or amphiregulin, respectively (12–14), and also induce mucin production from goblet cells. ILC2s were initially identified as cells that exist in dense fat-associated lymphoid clusters in adipose tissue, but later studies revealed that large numbers of ILC2s also reside in mucosal tissues, such as the intestine and lung. Moreover, recent studies have reported their presence in systemic tissues in both humans and mice, for example, brain, nasal polyps, salivary glands, tonsil, heart, liver, stomach, kidney, pancreas, uterus, skin, muscle, joints and peripheral blood (Fig. 1) (6, 11, 15–26).

ILC2s differentiate from hematopoietic stem cells via a common lymphoid progenitor and are generally thought to be derived from the bone marrow, because bone marrow-resident ILC2 progenitors exist and because bone marrow transplantation after irradiation replenishes ILC2s (1, 27). However, recent reports indicate that ILC2 progenitor cells already exist in peripheral tissues during the fetal period and that platelet-derived growth factor receptor α (PDGFRα)^+^gp38^+^ mesenchymal cells support ILC2 acquisition of type 2 cytokine production (28). Although this differentiation pathway appears to be essential for acquiring tissue residency and tissue-specific functions of ILC2s, it remains uncertain how ILC2 progenitor cells are supplied to peripheral tissues and become mature during ontogenetic development.

Over the last decade, regulatory factors for activation, suppression, expansion, development and migration have been reported for ILC2s; however, the source and the triggers of these factors are still unclear. Because most of the environmental factors are derived from tissue-constituting cells, the distribution and adjacent cells in tissues are important for the functions and physiological roles of ILC2s. To correctly understand the control mechanism of ILC2s, it is necessary to determine the potential effect of the surrounding microenvironment on ILC2 functions. In this review, we outline ILC2 tissue localization and explain the mechanisms of ILC2 regulation by surrounding cells to contribute to the development of basic research on ILC2s and the investigation of novel clinical targets for ILC2-associated disorders.

## Tissue distribution of ILC2s

### Role of ILC2s in the mucosal system

**Fig. 2.**
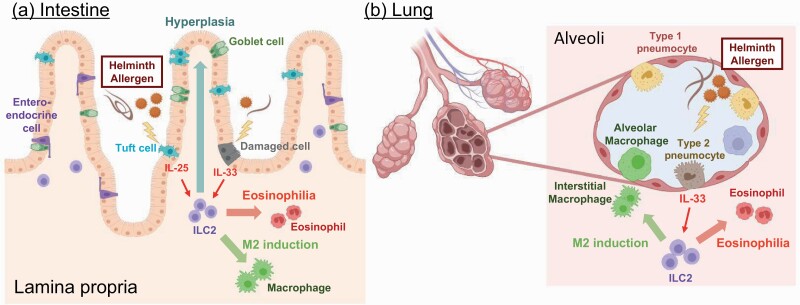
ILC2s are located adjacent to epithelial cells in mucosal tissues. ILC2s form dense populations in mucosal tissues, such as intestines and lungs. ILC2s are locally activated by IL-33 and IL-25 in response to tissue injury caused by helminth infection or allergen inhalation. (a) In the intestine, IL-33 is released from damaged epithelial cells, and IL-25 is produced by activated tuft cells to facilitate rapid ILC2 activation. (b) In the lung, type 2 pneumocytes are a major source of IL-33 to activate ILC2s. The ILC2 activation leads to eosinophilia, M2 macrophage induction, and hyperplasia of goblet cells and tuft cells, which contribute to helminth expulsion and to tissue regeneration.

Subsequent analyses revealed that ILC2s reside in various peripheral tissues, especially in mucosal organs, such as digestive organs and lungs (Fig. 2). In these tissues, ILC2s localize immediately below epithelial cells and are activated in response to epithelial damage by parasites or allergens. IL-33 released from necrotic epithelial cells or IL-25 produced from tuft cells activate ILC2s, leading to the induction of eosinophilic inflammation, M2 macrophage accumulation and enhanced mucus production (2, 29, 30). The process associated with the type 2 immune response is crucial for the expulsion of parasites or allergens and promotes the differentiation and proliferation of epithelial cells, thereby contributing to tissue repair.

### The ILC2 niche present in the perivascular adventitial cuff structure

**Fig. 3.**
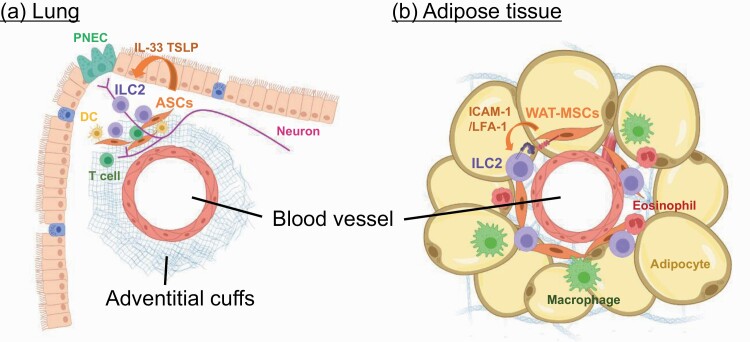
Stromal cells support the activation of ILC2s in perivascular regions. In various tissues, ILC2s localize with IL-33^+^ stromal cells in perivascular regions. (a) In lungs, ASCs associate with ILC2s, DCs or T cells within adventitial cuffs (collagen-rich structures) and support their activation by producing IL-33 or TSLP. (b) In adipose tissue, WAT-MSCs directly activate the proliferation of ILC2s via cell–cell interaction using ICAM-1–LFA-1. Consequently, ILC2s amplify the type 2 immune response in WAT by recruiting eosinophils or by enhancing the differentiation of M2 macrophages.

Recent reports show that ILC2s localize to the perivascular regions along with specific stromal cells producing ILC2-activating factors. Using three-dimensional imaging technology in IL-5 reporter mice, Dahlgren *et al*. (31) reported ILC2 localization in tissues, revealing that ILC2s accumulate in the outermost layer of blood vessels called the adventitial cuff structure that comprises collagen fibers and extracellular matrices (Fig. 3a). The perivascular adventitial cuff structure is present in various tissues, such as the lung, brain, liver, skin and lymph nodes, with dense pockets of ILC2s present in these tissues. The cuff structures comprise adventitial stromal cells (ASCs) as a major source of IL-33 and thymic stromal lymphopoietin (TSLP) and act as a niche for ILC2s both in the steady state and during type 2 inflammation.

Additionally, Rana *et al*. (32) identified white adipose tissue (WAT)-resident multipotent stromal cells (WAT-MSCs) as an ILC2 niche on the basis of their role as a reservoir of IL-33 in WAT and support of intercellular adhesion molecule-1 (ICAM-1)-mediated proliferation and activation of lymphocyte function-associated antigen-1 (LFA-1)-expressing ILC2s. Consequently, ILC2-derived IL-4 and IL-13 induce eotaxin secretion from WAT-MSCs to promote eosinophil recruitment to sustain a type 2 immune environment in WAT (Fig. 3b).

### Neuronal regulation of ILC2s in peripheral tissues

Recent studies revealed that ILC2s express high levels of receptors for several neurotransmitters, such as vasoactive intestinal polypeptide (VIP), neuromedin U (NMU), calcitonin gene-related peptide (CGRP) and norepinephrine (NE), and these factors regulate ILC2 activity in the steady state or during type 2 inflammation (11, 33–40). These reports suggest that the nervous system alters ILC2 function by a regulatory process distinct from that of cytokines. Additionally, numerous peripheral neurons extend to the intestinal tract and adventitial cuff structure, both of which are highly populated by ILC2s (31, 35, 39, 40).

**Fig. 4.**
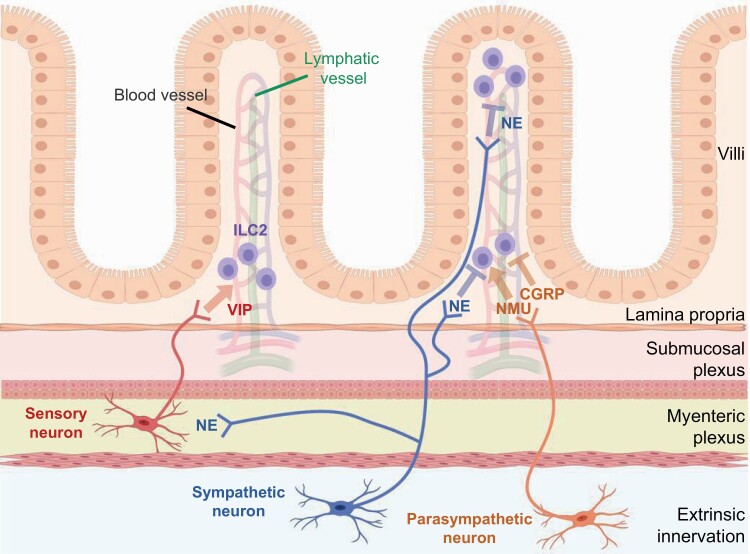
Various types of neurons reside proximal to ILC2s and regulate their function via the production of neurotransmitters. In the intestinal tract, sensory, sympathetic and parasympathetic neurons are involved in the regulation of ILC2s. Food intake stimulates sensory neurons to produce VIP, which induces constitutive IL-5 production from ILC2s. During helminth infection, NMU and CGRP from parasympathetic neurons and NE from sympathetic neurons affect ILC2 functions. NMU enhances the proliferation and cytokine secretion by ILC2s, leading to helminth expulsion. In contrast, CGRP and NE suppress cell proliferation and IL-13 production of ILC2s, which modulate ILC2 responses. Whereas sensory and parasympathetic neurons are enriched within the lamina propria, sympathetic neurons can be observed in both lamina propria and villi. These differences in both the effects of neurotransmitters on ILC2s and the distribution of neurons in the tissues can possibly contribute to the generation of the functional diversity of ILC2s.

Histologic analysis demonstrated that intestinal ILC2s localize with sensory and parasympathetic neurons in lamina propria, which release VIP and NMU or CGRP, respectively. Moreover, sympathetic neurons, which release NE, extend to both the lamina propria and villi and are adjacent to ILC2s during parasite infection (Fig. 4). Although studies of ILC2 activity related to nervous systems are in the early stages, further investigation might provide an answer to the remaining questions on allergic disorders, such as why asthmatic attacks are more likely to occur during sleep and why atopic dermatitis is exacerbated by stress.

### Dynamic migration of ILC2s

Under specific pathogen-free conditions, most ILC2s localize to peripheral tissues and are almost completely absent from lymphoid tissues, such as lymph nodes and spleen. Additionally, parabiosis experiments reveal that, unlike T cells in the adaptive immune system, ILC2s do not migrate to other tissues via the lymphatic system or blood circulation in the steady state (41).

However, IL-25 treatment reportedly promotes migration of inflammatory ILC2s, generally defined as IL-33 receptor ^low^/IL-25 receptor ^high^/killer cell lectin-like receptor G1 (KLRG1)^high^ ILC2s, from the small intestine to the lung or mesenteric lymph nodes during helminth infection (42). Huang *et al*. (43) demonstrated that intestinal ILC2s rapidly proliferate and enter the lymphatics and blood circulation in response to IL-25 and then reach peripheral tissues, such as the lung or mesenteric lymph nodes, in a sphingosine 1-phosphate-dependent manner. Moreover, another report showed that IL-33 stimulation altered the expression of chemokine receptors, such as C–X–C motif chemokine receptor 4 (CXCR4), on bone marrow ILC2s to promote their translocation, and some lung- and skin-resident ILC2s are thought to be supplied via this mechanism during the neonatal period (44).

Furthermore, although no ILC2s have been detected in bronchoalveolar lavage fluid (BALF) of naive mice, activated ILC2s exude into BALF during parasite infection or asthmatic inflammation. As intra-nasal administration of IL-25 or IL-33 can also recruit ILC2s into BALF without infection or allergen exposure, these cytokines potently mediate migration of ILC2s into BALF (41). Li *et al*. (45) demonstrated that C–X–C motif ligand 16 (CXCL16), which binds to CXCR6, is expressed in the airway during asthmatic inflammation, and IL-33 and CXCL16, but not IL-25, directly induce ILC2 migration *in vitro*. Even though the mechanism of these dynamic migrations of ILC2s is still unclear, the migration ability of ILC2s should be well considered to understand the source of ILC2s during type 2 inflammation.

## Cell-specific regulation of ILC2s in tissues

### Epithelial cells

Epithelial layers in mucosal tissues, such as the intestine and lung, are the first line of defense against parasites and allergens, with epithelial cells constitutively expressing IL-33 in the nucleus (46). As mentioned above, parasite- or allergen-derived proteases induce necrosis in epithelial cells, leading to the release of IL-33, and rapidly process it into a mature active form, which strongly activates ILC2s (47). Additionally, non-necrotic epithelial cells produce ILC2 activation factors, such as tumor necrosis factor (TNF)-like cytokine 1A and TSLP (48, 49). Moreover, some epithelial cells highly express E-cadherin, a ligand for KLRG1 whose expression is elevated on ILC2s as they get activated (26). E-cadherin–KLRG1 interactions reportedly suppress ILC2 functions and act as a negative feedback mechanism to suppress excessive ILC2 activation.

In addition to common mechanisms, some specialized epithelial cells, such as tuft cells and neuroendocrine cells, are involved in other forms of ILC2 regulation. Tuft cells reside in the epithelial layer of the intestinal tract, and bronchi have critical roles in ILC2 activation during the type 2 immune response by producing IL-25 (30). Tuft cells proliferate and produce IL-25 in response to ILC2-produced IL-13, thereby resulting in further activation of ILC2s in a positive feedback loop promoting type 2 inflammation. Additionally, recent reports have revealed that tuft cells initiate IL-25 production by direct recognition of metabolites or constituents of parasites (50). Specifically, nematode (*Nippostrongylus brasiliensis*) infection activates tuft cells and induces leukotriene C_4_ (LTC4) production, which activates ILC2s, in addition to IL-25 secretion, whereas protist (*Trichuris muris*) infection also activates tuft cells but only induces IL-25 production. IL-25 promotes GATA3-dependent activation, whereas LTC4 promotes nuclear factor of activated T-cell (NFAT)-dependent activation of ILC2s. Details of the specific roles of tuft cell-derived LTC4 in ILC2 activation remain to be elucidated.

Neuroendocrine cells are an epithelial cell subtype in digestive, respiratory and glandular organs that produce neurotransmitters and regulate the activity of the peripheral nervous system. Sui *et al*. (51) reported that pulmonary neuroendocrine cells (PNECs) in the lung localize with ILC2s at bronchi branch points and are associated with ILC2-mediated type 2 inflammation by CGRP production. Consistent with CGRP stimulation-mediated up-regulation of IL-5 production from ILC2s, PNEC-deficient mice demonstrate less ILC2 activation and asthmatic inflammation relative to control mice.

### Neurons

Many cytokines, neurotransmitters and lipid mediators are shared between the nervous and immune systems, and cross-talk between these systems is vital for maintaining tissue homeostasis. The nervous system comprises somatic and autonomic neurons that vary in their distribution and use of neurotransmitters. Recent studies reveal that ILC2s directly bind to neurotransmitters produced from various peripheral neurons, such as sensory, sympathetic and parasympathetic neurons.

Sensory neurons are responsible for transmitting sensory movements of the body and internal organs as signals to the central nervous system and are particularly abundant in the lung and intestinal tract. Most of these sensory neurons in lungs express nociceptors, produce VIP and exacerbate asthma symptoms by activating T cells and ILC2s (33). Additionally, sensory neurons of the intestinal tract produce VIP constitutively in response to food intake to contribute to IL-5 production by ILC2s during the steady state (11). VIP stimulation up-regulates cyclic adenosine monophosphate levels of ILC2s and induces IL-5 production. Because VIP production is enhanced by IL-5, this can create a positive feedback loop between sensory neurons and ILC2s during both steady and inflammatory states.

Sympathetic neurons, a part of the autonomic nervous system, are responsible for activating physical activity by promoting heart rate and vasoconstriction while inhibiting intestinal motility or digestive fluid secretion. They include adrenergic neurons that produce catecholamines, such as epinephrine and NE. Among the various adrenergic receptors, ILC2s selectively express β2-adrenergic receptor (β2AR) at high levels and its stimulation suppresses ILC2-specific proliferation and cytokine production (40). Histologic studies reveal that sympathetic neurons and ILC2s co-localize at the villi and submucosa of the intestinal tract, suggesting that the sympathetic nervous system prevents excessive activation of ILC2s. Numerous lymphocytes also highly express β2AR, with previous studies reporting that β2AR agonists affect the expression of C–C motif chemokine receptor 7 (CCR7) and CXCR4, which are involved in the suppression of lymphocyte egress into the bloodstream (52). It remains uncertain whether β2AR is also involved in ILC2 migration.

Parasympathetic neurons play an antagonistic role to sympathetic neurons and reduce heart rate, relax blood vessels and activate digestive activity, leading to a relaxed and calm state of the body. They are formed by cholinergic neurons and produce acetylcholine and NMU. ILC2s express NMU receptor 1, and its stimulation by NMU enhances IL-5 and IL-13 production from ILC2s in an NFAT-dependent manner (34–36). Histologic analysis revealed that ILC2s reside with vagal neurons, which innervate the lung and intestine as part of the parasympathetic nervous system. NMU production in vagal neurons is enhanced by stimulation via the IL-33 receptor and Toll-like receptors (TLRs) in an MyD88-dependent manner. Moreover, NMU-producing neurons recognize the invasion of parasites or microorganisms by sensing IL-33 or TLR ligands to promote ILC2 activation and enhance type 2 inflammation.

Interestingly, parasympathetic neurons also produce CGRP to suppress the expansion and IL-13 production by ILC2s during type 2 inflammation both in the lung and intestine (37–39). These bidirectional abilities of the parasympathetic nervous system may adjust ILC2s for proper functioning. Although interactions between ILC2s and the nervous system are not as pronounced as those associated with IL-33, elucidating these mechanisms will be important for understanding the behavior of ILC2s in diseases.

### Immune cells

There are many reports on the interaction of ILC2s with epithelial cells and neurons, but ILC2s also interact with immune cells. Various immune cells, including T regulatory cells (T_regs_), T helper 2 (T_h_2) cells and dendritic cells (DCs), localize with ILC2s in the perivascular adventitial cuff in various tissues (31). T cells, macrophages and granulocytes are found in close proximity to neurons, suggesting their possible interaction with ILC2s.

T_h_2 cells differentiate in a GATA3-dependent manner similar to ILC2s and contribute to type 2 inflammation as a major source of IL-4 and IL-13 in the adaptive immune response. Upon interaction with ILC2s, T_h_2 cells are activated by the OX40 ligand, programmed death-ligand 1 (PD-L1) and inducible T-cell co-stimulator (ICOS) ligand expressed by ILC2s, and this activation leads to the up-regulation of GATA3 expression and cytokine production (53, 54). Activated T_h_2 cells contribute to ILC2 activation by producing IL-4 or IL-9, with this positive feedback loop essential for orchestrating both innate and adaptive immune responses.

T_regs_ suppress other immune cells, including ILC2s, and exert anti-inflammatory effects. T_regs_ resident in adipose tissue in the steady state and induced in inflamed tissues during type 2 responses express high levels of the IL-2 receptor and IL-33 receptor, which further promotes the production of IL-10 and transforming growth factor-β (TGF-β) to suppress ILC2 function (55). Furthermore, T_regs_ express ICOS and produce IL-10 and TGF-β by direct interaction with ICOS ligand on activated ILC2s, resulting in ILC2 suppression (56).

DCs play an essential role in promoting the adaptive immune response and can be classified as either conventional (cDCs) or plasmacytoid (pDCs) on the basis of their differentiation pathways, marker expression and functions. Among cDCs, CD301^+^ cells (cDC2s) induce the type 2 immune response by producing IL-4 and IL-33 (57). Histologic analysis indicated that in the lung, liver and kidney, ILC2s frequently localize proximal to cDC2s in the perivascular adventitial cuff (31). Although interactions between cDC2s and ILC2s have not been clarified, a possible role involves ILC2 activation by glucocorticoid-induced TNF-related protein (GITR) ligand following IL-33-stimulation (58, 59). IL-33 up-regulates GITR expression on ILC2s, and GITR ligand stimulation subsequently enhances IL-9 production by ILC2s, resulting in autocrine activation of ILC2s. Antigen-presenting cells, such as DCs, express high levels of GITR ligand, suggesting that cDC2s might be involved in priming ILC2s by either IL-33- or GITR ligand-mediated cell–cell contact.

Unlike cDC2s, pDCs are a major producer of type 1 interferons, which suppress ILC2 activation (60). pDCs are present in small numbers in neonatal lungs relative to levels in adults and, consistent with these limited levels, neonatal lungs display higher levels of ILC2s accompanied by increased severity of asthmatic symptoms relative to adult lungs (61). Therefore, improvement of the function of pDCs may be a possible treatment strategy for childhood asthma. Furthermore, because DCs have a role as the major source of IL-27, which has suppressive effect on ILC2s, the interaction between DCs and ILC2s should be essential both for the initiation and resolution of type 2 inflammation (41).

A recent study revealed that ILC2s are regulated by various lipid mediators. Eosinophils and M2 macrophages, which infiltrate inflamed tissues following ILC2 activation, produce prostaglandin D2 and leukotrienes and may promote further ILC2 activation (62, 63). Additionally, basophils and mast cells are crucial cells associated with type 2 inflammation and produce cytokines and lipids, including IL-2, IL-4, IL-33 and prostaglandins to promote ILC2 functions (64, 65). In contrast, some myeloid cells are also important for ILC2 suppression. TNF-α production in adipose tissue following feeding with a high-fat diet promotes macrophage differentiation into an M1 phenotype along with up-regulated PD-L1 expression (66). These macrophages subsequently suppress cytokine production by ILC2s in a PD-L1-dependent manner, resulting in insulin resistance and metabolic dysfunction. Moreover, IL-33-stimulated mast cells initiate IL-2 production to promote regulatory functions of T_regs_, thereby preventing over-activation of ILC2s (55). A previous study involving live imaging of ILC2s on the skin reported their proximity and interaction with mast cells, suggesting a possible role for mast cells in functional switching of ILC2s (67).

Some factors produced by activated ILC2s perform autocrine or paracrine functions in ILC2s. IL-2 and IL-9 represent signal transducer and activator of transcription 5 (STAT5) activators important for ILC2 survival, proliferation and cytokine production (6). IL-33 stimulation promotes IL-2 and IL-9 production by ILC2s, which activates ILC2s in an autocrine manner. Additionally, IL-33 stimulation up-regulates both ICOS and ICOS ligand expression in ILC2s, with this interaction between ILC2s promoting STAT5 phosphorylation (68). In contrast, Nagashima *et al*. (38) reported that CGRP has a suppressive effect on ILC2s and that CGRP-stimulated ILC2s express high levels of CGRP by themselves, suggesting that ILC2s potentially suppress their own functions in an autocrine manner. Further investigation is required to elucidate the mechanisms involved in mediating cell–cell interaction between ILC2s.

### Other cells

ASCs and WAT-MSCs reportedly support ILC2 function in the peripheral microenvironment (31, 32). ASCs are present in various tissues and promote the formation of an ILC2 niche as a major source of IL-33 and TSLP. ASCs express high levels of collagen to create extracellular matrices and scaffolds for immune-cell interactions involved in ILC2 regulation (31). WAT-MSCs constitute a significant source of IL-33 and directly activate ILC2s via cell–cell interaction with ICAM-1–LFA-1 to promote proliferation and cytokine production (32). Because WAT-MSCs are also reported to reside around blood vessels, it might be possible that WAT-MSCs are a type of ASCs in WAT, which means that ICAM-1–LFA-1 interaction plays an essential role not only in adipose tissue but also in various tissues. Although such stromal cells are thought to produce chemoattractants that assemble immune cells, including ILC2s, T cells and myeloid cells, these mechanisms are poorly understood.

Additionally, recent studies revealed ILC2 involvement in anti-tumor immunity against solid tumors, such as those associated with melanoma and pancreatic cancer (9, 10). Although ILC2s accumulate around tumors and IL-33 administration activates ILC2s to reduce tumor size, tumors prevent ILC2 functions by several mechanisms. In a melanoma model, tumors producing lactic acid reduced the surrounding pH and suppressed ILC2 activation (9). Moreover, in a pancreatic tumor model, PD-L1-expressing tumors stimulated PD-1-expressing ILC2s and suppressed their function. Consistent with this finding, administration of IL-33 together with a PD-L1-neutralizing antibody increases ILC2 accumulation around tumors and reduces tumor size relative to administration of IL-33 only (10). These findings suggest that ILC2s exhibit anti-tumor activity; however, the mechanisms associated with this activity remain unknown.

## Conclusions

In recent years, increasing numbers of studies have identified novel factors associated with ILC2 regulation, and many reviews have summarized their molecular mechanisms. In this review, we outlined these mechanisms from the perspective of adjacent cells surrounding ILC2s in the tissue microenvironment to better understand the behavior of ILC2s in the physiological steady state and under inflammatory conditions *in situ*. Although many advanced studies have clarified the interaction between ILC2s and tissue-constituting cells, there are still unexplored questions concerning detailed mechanisms of ILC2 regulation.

ILC2s proliferate and produce cytokines via stimulation by IL-33, while simultaneously increasing the expression of receptors that suppress these activities (PD-1 and KLRG1). Besides, the expression of adhesion factors, such as integrins and chemokine receptors, associated with ILC2 migration and localization fluctuates significantly. These changes in surface molecules enable adaptation to the microenvironment in tissues and promote interactions that exert appropriate functions.

Interestingly, both IL-25 and IL-33 reportedly induce ILC2 migration across tissues, suggesting that processes related to localization in and movement between tissues coincide. A single-cell RNA sequencing comparing tissue-resident ILC2s revealed that the expression of cytokine receptors in ILC2s is quite different in each tissue (69). It has been shown that IL-33 receptor is highly expressed in the lung, adipose tissue and bone marrow; IL-25 receptor is highly expressed in the intestinal tract; and the IL-18 receptor is highly expressed in the skin. These differences suggest that crucial cytokines for ILC2 activation are used properly depending on each tissue, and the dependency on the cytokine is determined during the process of differentiation and maturation of ILC2s supported by tissue-specific stromal cells. Further analysis is needed to investigate the differentiation mechanisms of ILC2s with unique characteristics in each tissue and to determine whether migrating ILC2s from different tissues acquire a specific susceptibility to cytokines.

Studies on ILC2 localization have noted differences between humans and mice. Generally, peripheral blood is often used for human ILC2 research because ILC2s are detected even in healthy donors, whereas this is rare in the case of mice. Moreover, these studies reported that ILC2 number and function in peripheral blood reflect disease severity, with this used as an analytical index for estimating pathological conditions; for instance, in asthmatic patients ILC2s exhibit potent production of IL-5/IL-13 (70). Recently, a study comparing the abundance of ILC2s by performing a single-cell RNA sequencing in various human tissues showed that mucosal tissues, such as tonsils and intestinal tract, tend to be richer in ILC2s than non-mucosal tissues, such as the spleen and bone marrow, similar to mouse tissues (71). However, unlike the case of mice, there are few ILC2s in the lung, which is a mucosal tissue, and the abundance of ILC2s in the skin, which is a non-mucosal tissue, revealed differences in tissue-level localization by species.

Although ILC2s are considered to not have an antigen recognition receptor, human ILC2s derived from peripheral blood cultured *in vitro* express TLR1, TLR4 and TLR6; exhibit increased expression of CD154; and produce more IL-5/IL-13 in response to TLR ligands, thus enhancing B-cell activation (72). This report suggests that we have to consider the regulatory mechanism of human ILC2s, not only with the regulation by tissue-constituting cells but also with the direct interactions with foreign antigens. It is expected that humans will always be exposed to foreign antigens and the whole immune system will be activated, unlike mice that are kept under specific-pathogen-free conditions. This is a point that should be carefully noted as a difference between humans and mice.

ILC2s have been identified in various human tissues and are involved in many diseases according to either excessive activation (inflammation) or dysfunction (disrupted tissue homeostasis). Recent technical advances involving single-cell RNA sequencing should help elucidate the networks that control the optimal balance of ILC2 functions as we want, leading to the development of therapeutic strategies targeting ILC2s.
